# Towards a Sedimentology of Information Infrastructures: a Geological Approach for Understanding the City

**DOI:** 10.1007/s13347-017-0298-7

**Published:** 2018-01-15

**Authors:** Vlad Niculescu-Dincă

**Affiliations:** Institute of Security and Global Affairs, Faculty of Governance and Global Affairs, Leiden University, Turfmarkt 99, 2511 DP The Hague, The Netherlands

**Keywords:** Policing, Smart city, Infrastructures, Geology, Technological mediation

## Abstract

Drawing primarily on ethnographic research performed in a city in Romania, this paper provides a thick description of police practices and information systems in that municipality. It shows various ways in which technologies mediate policing practitioners’ perceptions, decisions and actions. Bringing some additional material from a case in the Dutch police in which they build risk profiles predicated on real-time data from a sensor network, the paper highlights new phenomena with ethical implications emerging at the intersection of information infrastructures and policing practices. In particular, it shows a solidifying effect of technologically mediated suspicion, a sedimentation process in technological infrastructures and the formation of pockets of prejudice in the layers of software code. To adequately account for these phenomena and others, the paper provides a set of arguments and conditions for a sedimentology of infrastructures. At the same time, it offers the first steps in a larger research project that would adapt and test the limits of a geological vocabulary and approach to understand smart urban environments.

## Introduction

Information infrastructures are becoming pervasive in urban environments—more so in some than in others—and are at the same time increasingly relied upon in policing practices. For instance, geo-spatial information systems are already a relatively widespread technology in many municipalities, engaged in both urban planning and crime mapping/crime analysis (Chainey and Ratcliffe 2005; Manning 2008); risk profiles are algorithmically generated entities often predicated on data from an array of sensors and increasingly engaged in resource allocation practices in policing (Schakel et al. 2013). Coming to grips with the role of networked information infrastructures in our urban fabric becomes even more relevant when we contemplate the significant investments in the development and roll out of ‘Smart Cities’ and similar agendas. On the one hand, such visions refer to knowledge-based economies, creativity and innovation, all made possible by smart people (Townsend 2013). On the other hand, a dominant feature of these visions remains a technology-driven emphasis on information infrastructures gathering and processing data to enable dynamic and efficient decision-making and fine-grained, real-time control (Hajer 2016, 52). In these visions, data tend to be relegated as neutral and objective, enabling measures that are free of political ideology regarding city life (Mayer-Schonberger and Cukier 2013; Kitchin 2014, 3).

In order to understand how infrastructures acquire their taken-for-granted character, how they lay claim to objectivity and what they do, a growing body of scholarly work has engaged in a type of archaeological work of ‘digging up’ the origins and consequences of bureaucratic and technological infrastructures (Bowker and Star 1999), showing the performative character of knowledge (Law 2009) and illustrating how artefacts—far from ‘purely technical’—can be highly moral and social (Latour 1988; Verbeek 2011). In time, scholarship in philosophy of technology and science and technology studies produced an adaptable vocabulary, ‘a list of terms, a set of sensitivities’ (Mol 2010, 253) for speaking about human and non-human assemblies and geographies of responsibility (Akrich and Latour 1992), accounting for the richness of user-technology interactions and the mediating role of technologies (Ihde 1990; Verbeek 2005).

This paper builds on and enriches this vocabulary to cope with the diversity of socio-technical phenomena related to the significant and projected expansion of information infrastructures, their thickening layers and their simultaneous ‘disappearance from the consciousness of the user’ (Gubbi et al. 2013, 1645). Broadening and deepening the archaeological focus on human activities in the past, this paper argues for a geological approach and vocabulary as a starting point for understanding our contemporary information infrastructures.

The paper develops its points with an analysis of a relatively widespread information infrastructure in municipalities and police organisations—geographic information systems (GIS) and their associated practices of data gathering, geocoding, mapping and analysis. In particular, it shows that even in the seemingly simplest and most common of these practices, information infrastructures are far from neutral intermediaries that produce objective renditions of reality but they play an active role, mediating the practitioners’ perceptions, decisions and actions. Bringing some additional material from a case in which the police use real-time profiles processing data from a sensor network, the paper shows how information infrastructures can accumulate prejudice in their code in a sedimentary process. At times, such pockets of prejudice rise to the surface in volcanic explosions, affecting persons, groups and communities.

The paper draws primarily on ethnographic observations I performed during July and August 2010 in the organisational arrangement of a local police in Romania, renamed in this paper M city.^1^ As typical for qualitative research, the study does not a priori claim to represent all the practices in that country as a whole but it highlights new phenomena by focusing on an in-depth exploration of the socio-technical arrangements in that organisation. The organisation was open to provide the requirement documents of the system and, due to the novelty of the system in their municipality, was able to provide recent insights in the changes and processes of shaping and working with their GIS.

This openness and availability allowed me to observe and map relations within the organisational, legal and architectural arrangements and between material configurations such as screens, cubicles and software-enabled entities. I gathered research data during the course of roughly 100 h of participant observation sessions in various situations. These included day shifts and night shifts in the control room, street patrolling with field agents, data introduction sessions with office personnel and participating in strategy meetings where police management made decisions based on GIS-generated maps represented on big screens. In addition to these observations, the analysis draws from internal police documents and system requirement documents that were made available to me by the local police management. All these allowed for my close observation of work processes and relations between police staff and the technological equipment they engaged with.

The paper is organised as follows. In order to understand the observations I made, I first give a brief overview of police reforms in Romania and present a set of characteristics of the organisational arrangements in the local police, including a few elements of the GIS design. After providing this context, I offer a starting point of the analysis with a vignette about the geo-coding of suspicion reports. In the following two sections, I draw on mediation theories (Akrich and Latour 1992; Latour 1994; Verbeek 2011) and analyse how the system mediates the officers’ perceptions of crime phenomena in the city as well as the potential consequences of this mediation when infrastructures accumulate sediments of prejudice. Drawing on these examples—and strengthening them with some additional material in another police force—I make an argument for a larger research project towards a sedimentology of information infrastructures.

## The Context of Local Policing in M City

Local police organisations in post-communist Romania were established in the mid 1990s as municipality services under the authority of local councils. They function separately from the centralised national police having as one of their aims to regain the legitimacy of the police, long eroded by the authoritarian ‘Miliţia’. As inspiration for their models, many municipalities looked for successful community policing implementation in western countries, following a general societal tendency towards western models.

In preparation for entering the European Union and NATO, and especially afterwards, this tendency saw an increasing synchronisation with newer models of policing and security strategies. One of these models that emerged along existing community policing models is Compstat, a combination of managerial, philosophical and technological arrangements for police organisations. It became an influential approach in policing since the mid 1990s after its association, albeit contested (Eck and Maguire 2000; Moore 2003), to significant crime reduction in the New York City Police Department. Despite its contestation, Compstat spread rapidly and became adopted in many police organisations throughout the world, being also implemented in the M city local police.

Compstat is a multi-layered approach, empowering and holding responsible police commanders to give rapid answers to crime problems in their areas. This is achieved by giving crime mapping and geographic information systems an important role in devising operational strategy (Ratcliffe 2008). In weekly meetings, ranking executives meet with local commanders to discuss emerging problems. Compstat is usually implemented to deal with controlling street crime, robberies, assaults or property crime, and analyses are based on geospatial data gathered from each area. Geographic information systems used in this approach basically include databases with spatially represented entities (e.g. crimes, incidents, suspects, weapons, groups) and software components that convert geo-coded data and superimpose it on the map of the city or the area. The information thus processed can be viewed on multiple layers. The images are able to cover the location patterns of a wide range of elements, from crime incidents, juvenile groups, offender activities to police patrol movements. In short, any element of interest in an area that can be represented spatially on a map can be included in a GIS.

In practice, the tasks of the local police organisation in M city involve activities such as preventing and combating street crime during demonstrations or public events; patrolling in parks and neighbourhoods; protecting buildings, monuments and other entities of public interest; identifying beggars or bringing homeless children to child protection agencies. Local police agents are responsible for handling contraventions and minor offences and delegate criminal offences to national policing agencies.

In this arrangement, they exchange information with the national police about suspicious behaviour pertaining to a wide spectrum of criminal occurrences that they encounter in their tasks of maintaining public order. The national police can potentially use the information exchanged with the local police in solving crimes or providing leads that were otherwise difficult to find. In a typical situation of collaboration, the local police patrol stops and identifies a person whose behaviour or presence at a particular place or time they assess as suspicious. The national police may then benefit from this information if that person turns out to be involved in a crime dealt with by them.

To accommodate this kind of collaborations as well as enable various Compstat-related crime analyses, the local police of M city implemented multiple changes in their geographic information system. A review I made of their internal police documents showed a number of 144 change requests since the initial deployment. The requests they made towards system developers range in complexity from correcting anomalies and fixing bugs to adding and modifying design features. For instance, one of the change requests concerned the addition of the category ‘suspect’ in the data introduction procedures, which was not available in the initial design. As one member of the police staff mentioned to me during an informal talk, this category was necessary as local police agents can stop and identify a person as a proactive strategy, aimed at deterring criminal behaviour.

Police tactics procedures specify that ‘the measure of interception’ applies both to those for which ‘there are clues to have committed crimes’ and also to those ‘assessed as suspect by police agents due to their presence at a particular place and time, their clothing, luggage or behaviour’ (Șerb 2006, 68). Upon such a stop and identification, the agents have to record the name of the person(s), the location and temporal data of the event, the offence they suspected the person of and what led them to this assessment. This information is noted in the report and later registered in the geographic information system. Afterwards, this data can be exchanged with the national police as part of their inter-institutional protocols or processed in crime mapping/crime analysis sessions.

## Studying Technologically Mediated Suspicion

When I began participant observation sessions in July 2010, local police agent Alexandra was working in the office as a data operator. She had the task to introduce the paper-based reports from field agents into the geographic information system. One of the observations I made during these sessions was that many reports arrived incomplete and ambiguous from field agents. Some arrived without the precise address while others lacked parts of the incident details. Agent Alexandra mentioned that the GIS requires a precisely defined location of events in order for her to place a ‘pin’ on the map in the process of geo-coding: ‘Look how sloppy they send the information. Here it’s just the street, no number, but the street is quite long. Here it’s between number 80 and 120, quite some distance. And here it’s ‘during the night of 28’ but the night is quite long, isn’t it? Anyway we put it in and for a strategic analysis is helpful.’

One type of reports caught my attention in particular as they contained only the note ‘susp.’ in the ‘details’ field, with no other specifications regarding the situation, reasons for assigning this label to a person or the type of offence. During the registration of such a note, after the entry of the date, location and names involved, the system displayed on the screen a drop down list containing types of offences. After a small moment, agent Alexandra chose ‘Theft’. Noticing the absence of this detail in the paper-based report, I asked agent Alexandra to explain her choice: ‘The program asks for an offence to be specified before I can go to the next step. Probably the suspect was searching through the trash bins as an alibi for stealing, probably bad clothing, kind of walking, hair style. What else could he have done in the parking lot at that hour?’ Agent Alexandra then closed the registration of the suspect report and moved on to the next one in the pile.

I remembered the name of the suspect. Later on, upon my request, another agent, Camelia, retrieved the data collected about that particular person. We found that he was a young Roma boy, age 14. The system retrieved 5 entries reporting that he was stopped and identified for wandering late. However, none of the entries described suspicion of theft or of any other penal offence except the last one. I asked agent Camelia her opinion about the person given what the system presented. ‘Obviously, a pickpocket (tr. pungaș). We’ve got to be very careful with them, especially when they hang around the touristic parts of the city’, came the response. I understood what her assessment was based on but I tried throughout the field research to understand this situation and its potential outcomes.

To be sure, this vignette depicts a situation representing only a potential first step in a long criminal justice chain with multiple possibilities for correction and adjustment. Moreover, assigning the attribute ‘suspect’ to a person in the local police system does not necessarily entail the procedure of detaining the person. As confirmed by several officers and management staff, agents are instructed that the local police system may contain ambiguities or plain errors. However, the vignette does show that in daily practice, the technological system mediated the agent’s perceptions (Verbeek 2011). As perceptions may influence their informal approach to persons, it is important to understand how this situation came to be.

The following sections perform a detailed analysis of this short vignette answering two sets of questions. One set concerns the outcome and implications of this situation: how did the system influence agent Camelia’s perception on the young boy? What implications can follow from this kind of technologically mediated practice? What happens when policing practitioners have only the system as their information source? To elaborate the answers, the section presents additional vignettes with situations in which practitioners worked in front of the big screens in the control room, analysing crime phenomena based on the GIS-generated output.

The second set of questions concerns the system design. What factors found their way in the technological infrastructure? How was the system configured? To answer these questions, the chapter introduces new observations that I made in the course of field research. They present different aspects related to the development and shaping of the information infrastructure in the M city local police. At the end of these two sections, the reader should have a richer tableau of the socio-technical arrangement in the municipality of M city.

### Mediating the Past

After retrieving the data about the young boy from the system, agent Camelia called him a ‘pickpocket’. She looked at the last entry in the system where he was classified a ‘suspect of theft’. It turned out that this entry influenced her view on the other entries in the system. She was inclined to quickly classify him in a category entailing an alerted attitude.

Of course, the situation was triggered by the interview question and it happened in the control room of the police station. If the retrieval of past data would have happened on the street, it is plausible that the agent would have the possibility to crosscheck the system with the information they acquire on the spot. Despite the influence of the system, agents could see and hear additional details at the scene of the incident. However, what happens with police opinions and perceptions of incidents or of crime phenomena when they only have the system, mediated by the screen of the computer, as the information source?

To explore the active role of technological infrastructures in shaping police perceptions, I will now present a set of vignettes in which police officers joined Compstat meetings in order to make strategic decisions based on GIS information. In preparation for these meetings, the main analyst was responsible for generating all kinds of thematic maps. For example, ‘maps with scandals’, ‘the distribution of beggars’ or ‘incidents with cars’ in a specific day/month/year. In order to identify patterns, she compared the maps with those exchanged with the national police. All these practices were meant to provide the police management with suggestions about possible next steps concerning resource allocation.

I sat with officer Roxana, the main analyst, during her preparation of such meetings. Working in front of her screen, officer Roxana was generally absorbed in intensive screen interactions. She often moved with the pen over the screen, looking at each indicator that produced a brief description of an incident/person.

After analysing the details of each incident, she took a broader view on the map of the whole city. She explained me the patterns she identified: ‘At the beginning of the year [2010] there was a boom in thefts. Probably from the crisis or something, they suddenly increased. Look here, first week a slight increase [Officer Roxana showed me multiple dots on the map circling them with the pen on the screen] and then, boom, almost all the city and it stays for several weeks. Then we reacted by sending agents in those areas and did identification just as it was during the time of Ceaușescu: Everyone after a certain hour was identified as suspect. Look here, it’s a week with a lot of thefts, nothing before, nothing after. It seems they tried the area but we were already alerted and sent patrolling squads.’

Afterwards, officer Roxana concentrated on a region in the city: ‘Look how this area gets formed. [She pointed with her pen to an area on the screen representing incidents] You see this hot spot? [She circled a bundle of dots on the screen with her pen]. It stays for several weeks until we intervene. [Then she showed me another map after their intervention] Look how they move after our actions [To illustrate this she produced several maps for consecutive weeks]. You can see how they cross the boulevard and move into this neighbourhood [At this moment she seemed to assume that the dots of incidents represent a group or a coherent criminal phenomenon acting in response to police patrol routes]. These established patrol routes look bizarre but they are not random. You could ask: why would we go at 2 o’clock in the night to this particular location? We decided to deploy patrols based on geo-location analysis showing the tendency of crimes to occur at these particular places and times.’

Officer Roxana made her analyses based on previously recorded data of geo-coded incidents. The quotes show a tendency of hers to rely on the system’s output and the previously introduced data. This is suggested, on the one hand, by the vocabulary and the tense of the verbs. She used the present tense to refer to the elements displayed on the screen as if they were direct representations of reality: ‘they *move*’, ‘look here, *it’s* a week of a lot of thefts’, ‘you can see how they *cross* the boulevard’. On the other hand, her reliance on the system is suggested by her vivid interaction with the screen. She pointed with her pen to areas on the screen as if they were the actual streets and districts of the city.

These quotes show that in her practice of interpreting the GIS-generated output, officer Roxana ‘black boxes’ whatever happened in the street as well as the reports of field agents and the office staff registrations. Their partiality, inadequacy or error become ignored in her practice. This analysis confirms what has been highlighted elsewhere, namely that GIS can often black box inconsistencies, misrepresentations or alterations of data (Graeff and Loui 2008; Jenkins and McCauley 2006) that are easily ignored in the daily practice of GIS users. Going about their daily routines, practitioners cannot frequently afford to assess the data quality or question the system’s output.

With these maps and analyses prepared, the analyst joined the Compstat meeting. Such a meeting typically gathered the chiefs of departments, the head of the local police and the main analyst. All were facing the screen wall, where the analyst presented the previously prepared maps. These contained each type of offence represented by coloured markers. For instance, car incidents in red, begging in black, street nuisance in yellow, etc. A separate map, on a separate screen on the wall, displayed the data that came in from the national police.

The head used these maps to identify broader spatial-temporal crime patterns in order to decide the next steps in resource allocation. At one point during the meeting, the head of police asked: ‘Why is that whole neighbourhood empty [of incidents], we used to have much more events there? Has it become so quiet?’ The response from the analyst followed: ‘Rather that we’re not there so much ... lack of motivation since the reductions [i.e. Romania implemented abrupt austerity measures in 2010, involving 25% salary cuts and 40% personnel reduction in public administration].’ The head responded: ‘Yes, that’s probably it. Next week we will make a special action in this neighbourhood on every offence [i.e. in order to compensate for the reduced presence].’

The meeting was focused on the screens, with the head of police asking for all kinds of reports and maps, comparing them with each other and with those of the national police. For instance, he requested a map with the incidents from the same month of previous years in order to try to identify trends over longer periods. At one point, he stared for several minutes, mentally being absorbed in the screens, with a silence that got the others stare at each other. ‘Show me what happened one week earlier’, said the head of police, breaking the silence. He requested a map which displayed the events that were geo-coded at the beginning of the same month of the previous year [i.e. 2009]. The analyst quickly generated the new map but did not remember any details regarding the displayed representations of events. The head of police looked at the screens and decided that all strategic orders and patrol routes stay the same.

These situations above highlight new aspects of the ways in which policing practitioners interact with technology. In this case, when the head of police doubts the apparent lack of offences in a certain neighbourhood, he allows the analyst to remind him of recent personnel reductions and demotivating salary cuts, better explaining what the system displays. When this sort of knowledge was not present (i.e. the analyst could not remember additional details from previous years), decisions relied solely on what the system displayed. In these situations, the system invited a particular kind of use, implicitly co-shaping the use that was made of it (Verbeek 2005). The officers’ perceptions of crime phenomena is mediated to a large extent by the generated maps. When officers cannot remember other details that would allow them to question the system’s output, their decision relies on whatever the system displayed. This is demonstrative of how technical artefacts *prescribe* dominant patterns of action (Akrich 1992). In this case, the ‘*script*’ of the technology defined a framework of action for the officers working with the system and this resulted in a situation in which they were inclined towards making certain decisions.

### Solidifying Suspicion

With the insights of this analysis, we can now return to the vignette in which agent Camelia classified the young boy. In a similar manner, she assessed the young boy as a pickpocket relying on the information that the screen displayed. The system ‘black-boxed’ what happened in the street and the translation processes afterwards. In this light, suspicion appears more than a mere social construct. Rather than forming in the heads of agents based solely on their sensory perception, it is also the system that performs what is a suspicious person or what crime phenomena/areas/times need more policing.

One insight of the mediating role of technologies concerns the influence they have on perceptions of reality and truth. Despite being aware of notoriously erroneous police databases—which many policing practitioners and scholars are—the evidence produced by the above sections supports the argument that technologies tend to ‘solidify’ suspicion. That it, they induce a perception of objectivity about the entities they represent as suspicious, problematic or risky. In other words, the very presence of a ‘suspect’ label into a technological infrastructure strengthens the officer’s state of alertness concerning the represented entity.

Of course, this does not mean that officers cannot and do not doubt the output of technologies or that this phenomenon occurs with every police officer and technological artefact they work with. Neither does it mean that suspicion implies arrest or conviction. Still, we have seen in the previous sections that technologies tend to ‘black box’ design decisions with normative charge or erroneous representations. As users, the police officers generally do not have access to and information about the criteria of design choices. In this way, these norms tend to become invisible, further ‘solidifying’ the police officer’s perception of suspicion.

Agent Camelia was not aware of the superficiality of the last registration in the system and she tended to take it for granted as an adequate indication of the character of the boy: ‘*Obviously*, *a pickpocket*’ was her assessment, based strictly on what the screen displayed. As she was in a difficult position to question the validity of system output, she took what was on the screen as a representation of reality, investing it with a high degree of objectivity. The last entry—the one where the boy was labelled as a suspect of theft—solidified her overall assessment.

Many information technologies in policing mediate the experiences and perceptions of practitioners in this way. With their assertive representations and powerful symbols, such as bright red icons, flashy alerts and high-pitched sounds, technology tends to be trusted. Police agents and officers work in fast-paced environments in which their behaviour is steered and nudged by a plethora of technological artefacts. These mediate their experience in the background or mediate their perception when officers embody artefacts in their practices. In these ways, technologies tend to induce a sense of objectivity and need of immediacy concerning the people and groups they perform as ‘suspicious’.

The insights from this section raise awareness of the paradoxical effect of technologically mediated policing to bring about a ‘solidification of suspicion’. On the one hand, software offers vast possibilities to capture the flexibility needed for the category ‘suspect’. On the other hand, software-enabled entities in policing tend to induce practitioners to rely on what is displayed on screens. Especially in proactive approaches, this phenomenon fosters a cycle of surveillance suspicion that people can have a hard time invalidating.

When technological infrastructures blackbox not only incomplete or ambiguous data but norms with ethical implications for whole categories of citizens, the potential for automated discrimination increases. The next section analyses this potential consequence of technologically mediated policing. In this case, some of the colour codes for offences encoded generalised views about categories of citizens in the configuration of the local police GIS.

### Sedimentation of Prejudice

As mentioned in Section 3.1, in the last text box, different colours represented offences on the GIS maps (red for car incidents, black for begging, yellow for scandals and so on). The officer responsible for choosing these colours at the point of configuring the system was officer Roxana, the main analyst. While her choices for most colour codes were apparently random, it turns out that her choice to use a dark colour for ‘begging’ was not. Upon asking her, the colour code expressed her view that begging, especially by children and youth, is practiced by the ‘dark skinned’ Roma ethnic group. As she was the officer responsible for configuring the system, her views got inscribed in technology (through the colour code). From that moment on, these multiplied in the maps with begging distributions throughout the city. Through the colour codes, her inscription entails a cumulative effect: begging is done by Roma ethnics.

I tested this effect by asking one officer his opinion about the way in which ‘begging’ was represented on the maps. His answer was: ‘I have no idea why they made it as it is [but after a moment]…probably from the skin colour…’. This answer, although triggered partially by the interview question, shows that the colour code encapsulated in the information infrastructure evoked a general perception of Roma. At the same time, without the interview question, the officer was not aware of the origins and action of this software-enabled entity. Its work was mainly beneath the surface.

Borrowing notions from geology, we might call this a ‘sedimentation’ process that takes place along the design and configuration of information infrastructures. In geology, the term sedimentation is used to describe the generic process by which particles that settle from a solution (e.g. water) come to rest and accumulate in layers at the bottom or against a barrier (e.g. on the river bed) (Nichols 1999). Sedimentary rocks are formed by the deposition of such particles called sediment. In this context, I want to suggest that infrastructures can be a site of similar processes of settling, sedimentation and lithification concerning configuration options, classifications, identity attributes, algorithmic steps or architectural decisions. In this case, the views of the police officer settled in the process of configuring the system and her choice accumulated along with others in the technological infrastructure. In other words, ‘the past’ settles in technological infrastructures and solidifies prejudice into ‘the future’.

Occasionally, such pockets of prejudice can become active and potent in mediating perceptions towards the enacted entities. In this case, not only was the GIS displaying one black dot on the configuration screen, as officer Roxana saw at the moment of configuring the system. Every weekly map with ‘the beggar distribution’ throughout the city visualises an eruption of black dots on the screens in front on the officers. As the other interviewed officer confirmed, the software-enabled maps evoked a cumulative effect. Irrespective of the statistics on the matter, the technological infrastructure induced a uniform perception of the phenomenon. The colour code had the potential to associate with the entire ethnic group, mediating the perception of the officer that worked with the system.

Staying within the sedimentation analogy, becoming infrastructural, design choices are covered by sediment and thereby invisible, and the prejudices become rock solid in the working routines of the local police. In this way, they can induce a perception of objectivity towards the enacted community, affecting their presumption of innocence. In its turn, a more alerted attitude increases the chance for encountering problematic situations with people in these communities (compared to others). Entering *a cycle of suspicion*-*surveillance* solidifies incentives for intervention and makes it difficult for the enacted entities to invalidate the reasons for which they raised police interest. Whereas in reactive policing, surveillance can work to invalidate suspicion; in proactive approaches, it tends to foster it.

The perspective developed in this analysis suggests that besides contributing to more efficient resource allocation, information infrastructures introduce their specific risks. We have seen in the previous section how the high-ranking officers made decisions based on the maps on the big screen. Both the head of the police and the GIS analyst presented a tendency to rely on what the screens displayed. The system prescribed decisions about the distribution of police patrols when the analyst did not remember any other details. Similarly, the analyst tended to rely on the screen when verifying individual incidents and persons or when identifying patterns.

This analysis suggests that it may not be sufficient to have a diverse workforce with respect to the staff’s background, covering multiple minorities. Once classifications sediment in infrastructures they tend to disappear from scrutiny and affect those communities implicitly. When infrastructures incorporate prejudice, it does not matter that much how well-intended are the individual officers as they work with technologies that actively mediate their perception and action. Classifications in police systems concerning ‘problematic’, ‘suspected’ or ‘risk’ entities can be sufficient justifications for an alert attitude or for engaging in proactive surveillance. In these ways together information infrastructures can silently but effectively contribute to eroding the trust between communities and the police when they carry hidden discriminatory potential.

### The Depth and Variety of Sediments

Notions of sedimentations and depositions from geology invite reflection over strata and layers as well as the nature of the sediment. We can think for instance that layers of sediment can range from the easily accessible, close to the surface, to the deeper and harder to access strata. We can also think of the diverse nature of the particles that come together and make up the sedimentary rock. For instance, in the above example, one might argue that the configuration option of the colour codes is easily accessible from a software perspective. A stratigraphy of this information infrastructure might classify the above sediment as belonging to a relatively superficial stratum with respect to the ease of access. At the same time, pockets of prejudice can also form in deeper strata of the software stack. That is, police information infrastructures can include outdated or prejudiced information directly in the code that processes data from a variety of sensors distributed in the environment. Similarly, the nature of the choice that ended up in the software of the information system pertained in this case to ethnicity, whereas many more identity attributes and design choices could become part of infrastructures.

The additional empirical material that supports these points comes from a case study in the Dutch police that has been analysed in more depth elsewhere (Niculescu-Dinca 2016, 127). In that project, the police engaged with risk profiles based on real-time traffic data coming from a network of automatic number plate recognition cameras. For instance, the following quote from an interview with a profile developer shows how risk criteria are defined and were considered for incorporation in a profile to capture potentially illicit activities: ‘We connect to RDW (Dutch vehicle registration database) and we get all the information about the car, the make, the colour, the fuel type that it uses, but also on the owner of the car. In this way, we can, for instance, say we want to stop all the cars which are very expensive, newer than 3 years and the license plate holder, the owner, is younger than 27 years old. We find it interesting that someone who is quite young still has a brand new car or almost brand new car that is so expensive. Where does he get the money? So we make a profile on this and check if this is a new car, if it is so expensive, and if the owner is younger than 27. If this is all true, then we say: you give a hit’. On the one hand, this quote highlights how profiling can combine a wider diversity of identity attributes (in this case name and age) that are used in the code of an aggregated profile together with data from an array of sensors. On the other hand, these algorithmic choices are only accessible to the programmers in the police organisation and can be changed only by modifying the code and recompiling the software module.

This kind of sediments can also form pockets of prejudice, meaning in this case that they can entail discriminatory outcomes when those areas of code get activated, and are at the same time more difficult to access than configuration options. In this context, the threshold of ‘27 years old’ would effectively put under surveillance not only illegal activities but, for instance, a whole category of young, successful entrepreneurs who can afford expensive cars at that age. Such real-time profiles predicated on widely distributed sensing infrastructures can trigger automated alerts on a variety of criteria, while their translation into profile code can be fraught with normative charge. When is someone old enough to be out of suspicion for owning a particular kind of car? Through connections to persistent databases and other sensor networks, the assemblage may put under surveillance larger categories, groups or areas.

These considerations render the profiles’ code as a locus of ethical investigation as their problematic character depends on algorithmic conditions, Boolean operators, particular values given to parameters and a wide variety of identity attributes. Getting combined in code, these attributes become tightly packed together in the software stack and thus they are not best understood as isolated features of identity but as shaped and realised in complex webs of identity and social relations (Hoffmann 2017). These accumulations trapped in various layers of the software stack trickle down invisibly in the broader socio-technological arrangements and, unchecked, can become lithified and potent.

### Partial Conclusion

On the one hand, the analysis of the situations above points to the continuing need for transparency in data-processing practices. While taking into account the specific nature of policing activities, the right of access to personal data of citizens to be able to correct information remains of particular emphasis in the future implementation of the General Data Protection Regulation. In this sense, this paper supports policies that recommend organisations to ‘clearly describe their subject access procedures’ and ‘provide explicit protocols for submitting an access request’ (Norris et al. 2015, 3). While not necessarily representative for all police organisations and information infrastructures, the findings above highlight a combination of phenomena that can yield problematic situations for individual persons and categories of citizens when (inaccurate, erroneous or biased) data is interpreted in a remote context as an objective representation of reality.

On the other hand, the analyses of the previous sections point towards the need for ongoing investigations of socio-technical infrastructures. Problematic situations can occur without the awareness of data subjects and even without police officers’ intention or awareness. Practitioners assess persons, areas or situations not only with their eyes and their minds—acting on evidence or on the cultural assumptions they are making—but suspicion can be embedded in code and displayed on screens. And, as we have seen above, data remains prone to partiality, inadequacy and bias, also when it is mediated by technologies. Rather than being objective representations of reality—justifying surveillance or supporting prosecution in the criminal justice chain—technologically mediated suspicion appeared here quite questionable.

Of course, a way to tackle many of these issues is by raising awareness also among the programmers and designers of infrastructures. This can be done by constantly and iteratively questioning the design process and reflecting on a wider range of issues and identity attributes, in what Wittkower calls ‘diversity impact assessments’ (Wittkower 2017). Such assessments should account not only for issues of ethnicity or age, as discussed above, but also for a broader set of issues including gender, (dis)ability, race, religion, nationality and their intermingling in complex socio-technical infrastructures. The outcome of such processes should not only identify the ways in which design choices may impact people, groups, areas or communities but also actively try to minimise it.

Still, more often than not problematic aspects of infrastructures remain buried and hidden. They work under the surface, eroding, accumulating or carrying with them digital debris. Neither data subjects, nor practitioners and designers may be aware or able to mitigate and minimise them. Especially in a thick mesh of technical and social infrastructures and large data flows that characterise visions of ‘smart cities’, many problematic phenomena may or may not be easily traced back to a design choice. In order to research and address such kind of problems, we may, indeed, have to ‘pierce the veil of technological wonder’ (Hoffmann 2017, 5) with a much stronger and thorough approach and vocabulary than what archaeology enables us to do. In short, we may need to be ready to drill rather than just dig.

## Towards a Sedimentology of Infrastructures

On the one hand, archaeology—as one of the first academic disciplines to seriously analyse technological change and the social role of artefacts—has a major contribution to this day in the spread of technological determinism (Wyatt 2008) with its intellectually poor and politically debilitating assumptions (Bijker 2010). Wyatt, drawing on Mumford, shows how our contemporary tendency to give reductionist accounts for the social role of technology often stems from an archaeological vocabulary that associates societies, civilisations or whole ages to a single material artefact that happened to be the remaining record (e.g. ‘Stone age’, ‘Bronze age’, ‘Iron age’ or more recently ‘Computer age’, ‘The age of Big Data’, ‘The age of social media’). As Mumford argues, ‘the absence of documents and the paucity of specimens resulted in a grotesque overemphasis of the material object, as a link in a self-propelling, self-sustaining technological advance, which required no further illumination from the culture as a whole […]’ (Mumford 1961, 231). In turn, the technologically determinist habit in vocabulary persists and pervades a whole range of contemporary institutions such as ‘museums, schoolbooks, newspapers […] television and radio’ (Wyatt 2008, 168). Derived from archaeology, this kind of vocabulary tends to explain complex socio-technical phenomena by identifying the man-made artefacts as the primary driving factor. In this sense, a rich geological vocabulary to the research of information infrastructures would be a step towards correcting this unfortunately old linguistic habit.

Firstly, notions from geology proved useful to increase the scope of analysis of information infrastructures in both breadth and depth. Just as archaeology, geology retains an approach of ‘digging up’ strata while its scope is much wider and deeper. Its contribution ‘gently shifts the existing theoretical repertoire. And then, as the theoretical repertoire shifts, it becomes possible to describe further, different cases, and to articulate so far untold events (relations, phenomena, situations)’ (Mol 2010, 261). We have seen throughout this paper how to understand processes of settling, sedimentation or lithification in the layers of information infrastructures.

Secondly, a *sedimentology of infrastructures* might prove a more adequate starting point of investigation without assuming particular distributions of responsibility between the role of humans and non-humans. Of course, human activity plays various roles in design processes and, at the same time, geology did recently expand its vocabulary to account for human activities and their impact on the earth’s geology with terms such as the ‘Anthropocene’ (Crutzen and Stoermer 2000). Still, to be able to identify and account for the kind of phenomena that may form in our information infrastructures, we may need to be ready to account for a whole set of processes that may or may not be traced to an initial human activity. Sedimentology studies the structure of sedimentary rocks and the processes involved in their formation (Nichols 1999). When geologists engage in studying sedimentary layers with this approach, they may, of course, discover traces of human activity along the way (and collaborate with archaeologists) but they do not a priori look for anthropocentric explanations.

Thirdly, sedimentology offers a set of principles to understand and interpret geologic history through observations of sedimentary structures. Such principles can be adapted and applied to the study of information infrastructures. For instance, *the principle of lateral continuity* specifies that layers of sediment initially extend laterally unless obstructed by a physical object or topography (Nichols 1999). As a result, sediments that are otherwise similar but are found separated (either by a new formation or by the result of erosion such as a riverbed) can be assumed to have formed an initially continuous layer. Adapting this principle to the study of information infrastructures suggests that the discovery of a sample from a sediment of prejudice rises the reasonable expectation of finding more similar sediments upon further investigation in the same layers of the software stack. The layered nature of software architectures allows for the adaptation and application of these principles that have been developed in geology to analyse how layers of sediment form and change through time and space.

Fourthly, a geological metaphor to research infrastructures may conjure up not only an image of solidified layers and static entities but also an image of dynamic processes such as *flows* of *debris* or *volcanism*. We have seen in this paper for instance dynamic phenomena when we analysed the generation of weekly maps with the beggar distribution. Such geologic metaphors can already be found in the literature that deals with the interplay of digital technologies and society. For instance, Haggerty and Ericson speak of the trails of information about a person’s habits and lifestyle as ‘the *detritus* of contemporary life’ (Haggerty and Ericson 2000, 611). Bowker and Star (1999) briefly hint themselves at geology in their search for ‘a new set of metaphors linking traditional social science and computer and information science’ to account for ‘the fluid dynamics of how classification systems meet up – a plate tectonics rather than a static geology’ (Bowker and Star 1999, 31). Therefore, unifying geological metaphors offers a rich vocabulary, notions and principles to research a myriad of phenomena with various degrees of dynamism, structure, evolution or depth.

Fifthly, developing a geological approach to understand technological infrastructures becomes even more relevant in a context in which many cities and organisations engage in practices of data ‘mining’—yet another metaphor closely related to the enterprise of understanding and exploring sediments. The vocabulary of many organisations is already pervaded with data-mining practices in which analysts seek to ‘extract’ patterns of behaviour that may be ‘hidden’ within large ‘piles’ of data.

On the other hand, one should keep in mind that analogies and metaphors are useful in as much as they expand and enrich our thinking and help us come to grips with new phenomena in a fruitful way but we should always be aware of their potential limitations. In this sense, the current paper offers only the first steps in a larger and ongoing research project that engages the fusion between geology and technology. While adopting and testing the limits of a geological vocabulary and approach to the research of infrastructures, we must be careful not to allow technological determinism to lurk beneath the surface. Even if notions such as the ‘Anthropocene’ begin to account for human activities in the geological vocabulary, infrastructures should, of course, not be understood as purely natural processes developing outside the influence of social, political and ethical considerations. Rather, the argument of this paper is that in order to thoroughly research the thick layers of our socio-technical infrastructures, we would be better off by taking as a starting point the mind-set and the vocabulary of the geologist.

## Conclusion

This paper focused on the practices around a geographic information system of a local police organisation in a city in Romania with some additional material from a sensor network project in the Dutch police. It followed the policing practitioners in their daily routines of designing and working with information infrastructures along organisational structures, legal frameworks, urban space, citizens’ behaviour and their formal and informal arrangements. The analyses do not claim to represent all contemporary technologically mediated practices, police organisations, let alone whole city infrastructures or police systems. As specific for qualitative research methods, they provide in-depth explorations of socio-technical ensembles, illustrating, highlighting and contrasting (new) phenomena. In this sense, they act as starting points, arguing for and pointing towards further research of technologically mediated practices with the proposed theoretical approach and vocabulary. In other words, I have just scratched the surface*.*

Through vignettes developed from observations and interviews, the paper first highlighted partial and ambiguous registrations in the geographic information system. Drawing on a constructivist vocabulary and theories of mediation (Latour 1994; Verbeek 2011), the analysis illustrated how the city information infrastructure performed entities and actively mediated the officers’ perception of persons, areas or crime phenomena, prescribing a more alerted attitude towards particular individuals and groups. In this light, ‘suspicion’ appears more than a social construct. Rather than forming only in the minds of practitioners—based on hunches, established facts, culturally shaped categories or prejudice—a suspect person, group, area or behaviour is also what the infrastructure enacts as such.

One effect we have seen is a *solidification of suspicion*. Once a suspect in a proactive policy is ‘in the system’, it becomes prone to have this status maintained and strengthened. Of course, in policing practice, the suspicious entity can prove to be the actual culprit and, in hindsight, the surveillance proves justified. Still, we have seen throughout this paper that suspicion remains prone to partiality and inadequacy, also when mediated by technologies. Rather than offering objective representations of reality, the evidence of this paper supports the point that (city governance) technologies enact particular versions of reality while simultaneously ‘asserting a particular expression of power/knowledge’ (Kitchin et al. 2015, 24).

At the same time, rather than solely determining outcomes, the infrastructures were shaped within a heterogeneous set of technical and social factors. For instance, the colour codes of offences were configured by the main analyst and her views got trapped in the infrastructure. In this way, the socio-technical ensemble prescribed a generalised view of the Roma ethnic group for the officers working with the system in the city’s local police. Bringing forth additional examples from the use of a sensing infrastructure in real-time profiling of road traffic, the paper showed how infrastructures can encapsulate pockets of prejudice between their layers. These accumulations tend to acquire a character of objectivity and become simultaneously potent and invisible. Lithifying ‘the past’, infrastructures can carry on prejudice into ‘the future’.

Drawing on the analysis of these phenomena, the paper derived conclusions on two levels. On a legal level, it reiterated the importance of transparency in data-processing practices and the need to empower data subjects to effectively exercise the right to access to personal data. On a methodological level, the paper proposed a geological approach to the study of information infrastructures. It provided a set of arguments and conditions for a sedimentology of infrastructures and proposed the development of a larger research project that would adapt and test the limits of a geological vocabulary and approach to understand smart urban environments.

This means developing a stratigraphy of software layers of urban information infrastructures and then beginning to excavate in loci such as the criteria, algorithms, categories and values that shape their design and architecture. Without fetishising the importance of code, failing to engage in a *sedimentological approach* allows for the formation of pockets of prejudice in the layers of urban information infrastructures. Unifying geological metaphors and expanding them to information infrastructures offers a rich vocabulary, notions and principles to account for a diverse set of processes with various degrees of dynamism and potential: accumulations, depositions, erosions and also flows, explosions, volcanism and more. In other words, we may need the mind-set, vocabulary and helm of the geologist to investigate our increasingly thicker urban information infrastructures.

## Footnotes

1 To protect the anonymity and confidentiality of officials and police staff, the name of the city has been turned into M city. The same applied to the names of the GIS technology company and police staff throughout the paper. M city is an important economic, industrial and cultural centre of Romania. It has ~150 thousand inhabitants with a mixed ethnic structure.
